# Correlation between two-dimensional micro-CT and histomorphometry for assessment of the implant osseointegration in rabbit tibia model

**DOI:** 10.1186/s40824-021-00213-x

**Published:** 2021-04-13

**Authors:** Hao-Zhen Lyu, Jae Hyup Lee

**Affiliations:** 1 Department of Orthopedic Surgery, SMG-SNU Boramae Medical Center, Boramae-ro 5-gil 20, Dongjak-gu, Seoul, 07061 South Korea; 2 Department of Orthopedic Surgery, College of Medicine, Seoul National University, Seoul, South Korea; 3 Institute of Medical and Biological Engineering, Medical Research Center, Seoul National University, Seoul, South Korea

**Keywords:** Bone-implant interface, Histology, Osseointegration, X-ray microtomography

## Abstract

**Background:**

Histology is considered as a gold standard for analyzing bone architecture. However, histomorphometry is a destructive method and only offers the bone information of a limited location. Micro-computed tomography (μCT) is a non-destructive technology and provides a slice at any site. The aim of this study was to compare the correlation of the Bone-to-Implant Contact ratio (BIC) between 2D micro-CT (μCT) and histomorphometry and to investigate a method for assessing the osseointegration of the implant by 2D μCT.

**Methods:**

A total of 18 implants were divided into three groups (6 implants per group), and inserted into the rabbit tibia defects as follow: implant only (Implant group), implant with β-TCP/hydrogel (TCP group), implant with rhBMP-2 loaded β-TCP/hydrogel composite (BMP-2 group). After 4 weeks of implantation, the specimens were collected to take the micro-CT scan with an aluminum filter and performed H&E staining on the undecalcified sections. The 2D μCT slices were chosen at an angle of 0°, 45°, 90° and 135° with the representative histological section to measure BIC. And the correlations between BICs of 2D μCT and BICs of histology were evaluated.

**Results:**

In each group, BICs at the same sites measured by histomorphometry and corresponding 2D μCT presented the same trend and shown no significant difference between the two methods (*P* > 0.05). BICs of histological sections and BICs of corresponding 2D μCT slices presented a strong correlation in the implant group (γ = 0.74, *P* = 0.09), a moderate correlation in the TCP group (γ = 0.46, *P* = 0.35), a weak correlation in the BMP-2 group (γ = 0.30, *P* = 0.56). In the implant group, the relationship between BIC-Mean-μCTs and BICs-Histology has presented a significant linear correlation (γ = 0.84, *P* = 0.04).

**Conclusions:**

Integrating bone information of several 2D μCT slices in different sites to measure BIC is a feasible method for assessing the implant osseointegration.

## Background

In dentistry and orthopedic, the osseointegration of the implant is important to assess the stability of internal fixation. Bone-to-Implant Contact ratio (BIC), the ratio of the length of bone direct contact to the implant thread to the length of the implant thread, is measured for evaluating the osteointegration and stability of the implant [1–3].

Currently, histomorphometry is considered as the gold standard for analyzing the BIC [4]. However, the making process of the histological slide is tedious, time-consuming, and destructive. Only a few representative cross-sections in the specific position of the implant is obtained by histological section and insufficient to provide the overall information of the implant [4–6]. Additionally, the specimen after the histomorphometry cannot be used for the other assessments.

Due to time-saving, convenience and nondestructive, micro-computed tomography (μCT) has been extensively used to evaluate the structure of bony tissue. The μCT dataset could be reconstructed to observe the bone architecture in any location around the implant and analyze the parameters of bone in the region of interest (ROI). However, because of the different attenuation coefficients of bone and implant in the specimen, metallic artifacts are generated on the bone-implant interface by the metal implant [7–9]. Therefore, the artifact affects the BIC obtained using μCT. Some previous studies recommend using soft filters to reduce artifacts during CT scanning, such as an aluminum or brass filter [10, 11], and using the correction functions of the analysis software during CT reconstruction to decrease the interference of artifacts, such as misalignment compensation, ring artifacts reduction, and beam-hardening correction [12]. Furthermore, a few studies suggested measuring BIC after excluding several voxels close to the screw surface to eliminate the artifact zone [13–15]. At present, there is no ideal method or program available for measuring BIC by μCT [16].

So far, the BICs have been obtained using the two-dimensional (2D) μCT images or the three-dimensional (3D) μCT models by different methods. In these researches, authors detected the correlation between the BICs of μCT and the BICs of histology for investigating the feasibility whether the BIC-μCT could replace the BIC-Histology to assess osseointegration [11, 17, 18]. However, the results of the correlations between the two methods were various in these studies. There were few studies to evaluate the osseointegration of the implant by integrating 2D μCT slices of multiple locations [6].

Due to the insufficient amount of autografts for treatment, synthetic bone substitutes that include bone graft-based substitutes (allograft, xenograft), ceramic-based substitutes (hydroxyapatite, calcium phosphate), growth factor-based substitutes (BMPs, PRP, FGF), and their combinations are used to promote bone regeneration [19, 20]. The development of new bone substitutes need to evaluate the osteogenesis efficacy in vivo experiment. If the implant osseointegration could be assessed by micro-CT, it saves time and cost.

In this study, we loaded recombinant human bone morphogenetic protein-2 (rhBMP-2) on the β-tricalcium phosphate (β-TCP)/hydrogel composite and inserted it with the dental implant into the rabbit tibial bone defect model to promote bone formation [21, 22]. We measured the BICs on the 2D μCT images at different sites of the implants using the manually measuring method that was performed in the histomorphometry. The aim of this study was to compare the correlation of BIC between 2D μCT and histomorphometry and to investigate a method for assessing the osseointegration of the implant by 2D μCT.

## Methods

### Preparation of rhBMP-2 loaded hydrogel composite

The β-TCP microspheres (Cerectron Co., Korea) were made by a spray-dry method for spherical particles in a 45–75 μm diameter range. The poloxamer 407 (BASF, German) at 18–22% concentration presents a sol-gel transition under 25 °C and exhibits the gel status at 37 °C. The 0.4 g β-TCP microspheres, 0.4 ml poloxamer 407 hydrogel at 35% concentration and 0.3 ml of 1.33 mg/ml *E.coli*-derived rhBMP-2 (Daewoong Pharm. Co., Korea) solution were mixed in situ that we previously described [23, 24]. The final concentration of rhBMP-2 in the hydrogel composite was 50 μg/ml.

### In vivo

Three New Zealand white male rabbits (3–3.5 kg) with no disease signs were used for the animal experiment in this study. The rabbits were raised in the standard cages and had an acclimation period for at least 1 week before surgical procedure. Zolazepam-tiletamine (Zoletil®, 15 mg/kg, Virbac Co., Korea) and xylazine hydrochloride (Rompun®, 7.5 mg/kg, Bayer Ltd., Korea) were intramuscularly injected for the general anesthesia. A skin incision was made on the anteromedial of the tibia along the longitudinal line. The fascia and periosteum on the tibia shaft were removed to expose the cortical bone. Three round defects (4 mm diameter) were drilled in a tibia shaft, and the distance between each defect was at least 12 mm to avoid interacting in osteogenesis. The same surgical procedure was performed on the bilateral tibia of the three rabbits. A total of 18 defects were randomly divided into three groups (6 implants per group). The dental implants (Ø 4 mm, 8.5 mm length, MegaGen Co., Korea) inserted into the rabbit tibia defect models as follow: Implant group (implant only), TCP group (implant with 0.1 ml β-TCP/hydrogel), and BMP-2 group (implant with 0.1 ml β-TCP/hydrogel loading 5 μg rhBMP-2). In the TCP group and BMP-2 group, the 0.1 ml β-TCP/hydrogel or 0.1 ml β-TCP/hydrogel contained 5 μg rhBMP-2 was placed in the defect by syringe, and then the dental implant was inserted into the bone defect. The three defects of each tibia belong to the same group. The cefazolin (300 mg, Yuhan Co., Korea) was intramuscularly injected 2 days for antibiotic therapy. After 4 weeks of implantation, the implant specimens were harvested to perform the micro-CT scanning and histology analysis. This study was permitted by the Institutional Animal Care and Use Committee (IACUC No. 11–0231) in the Clinical Research Institute of Seoul National University Hospital.

### Micro-CT processing and analysis

**Fig. 1:**
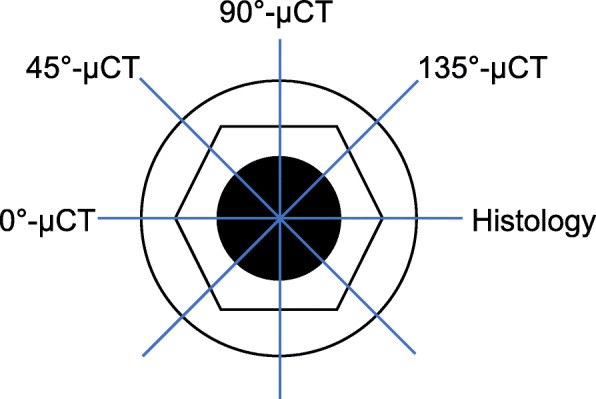
Sites of the micro-CT slices and the histological section for BIC measurement. The 2D micro-CT slice, named 0°-μCT, was corresponding with the representative histological section. The 2D micro-CT slices at the interval of 45°, 90°, and 135° from the histological section, named 45°-μCT, 90°-μCT, and 135°-μCT, were selected to calculate BIC

**Fig. 2:**
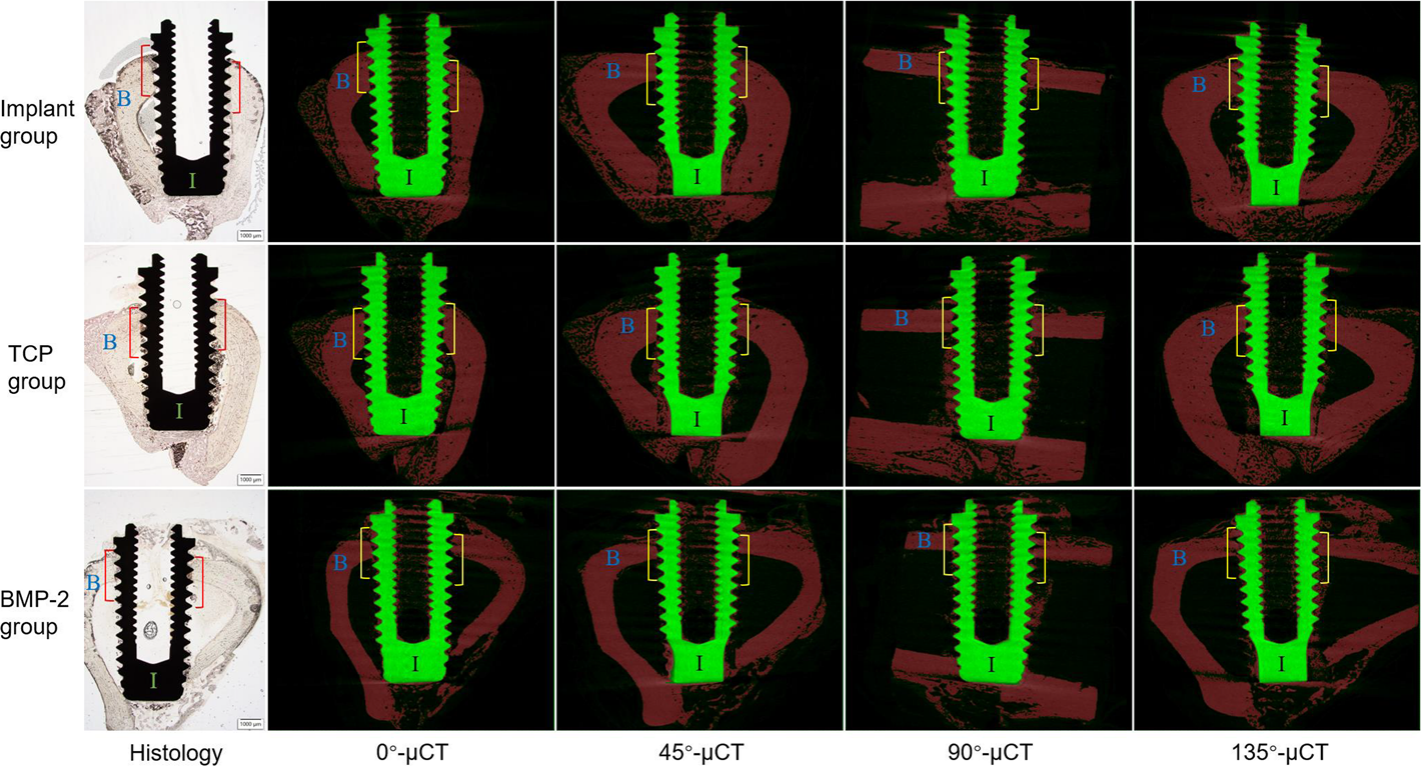
Histological section, and the four 2D μCT slices at different sites of the implant. The four grooves on both sides of each slice were chosen to measure BIC for assessment of implant osseointegration in the three groups. Histology: H&E staining, bar = 1 mm. B: bone tissue; I: implant

Each harvested tibia was divided into three specimens containing implants. The specimens were scanned using the Skyscan 1173 micro-CT (Kontich, Belgium) with a 0.5 mm aluminum filter at 60 μA, 130 kV and a resolution of 20 μm. The micro-CT raw dataset was reconstructed by the NRecon Software (Version 1.5.1.1, Skyscan, Belgium) with ring artifact correction at 5 and beam hardening correction at 40%. According to the geometric markers of the implants and host bone, the 2D μCT slices matching with the representative histological slices were acquired by the Dataview Software (Version 1.4.0.0, Skyscan, Belgium) (Fig. 1). Next, the optimal threshold (min 60, max 90) of the newly formed bone around the implant was identified in micro-CT using the corresponding histologic sections as a reference. The 2D μCT binary images, then, were converted to halftone images to distinguish air and soft tissue (blank), bone (red) and implant (green) with the CTAnalyzer Software (Version 1.12.0.0, Bruker, Skyscan) (Fig. 2). The upper four grooves in each side of the implant on the 2D μCT slices were chosen to measure BICs by the Image J Software (US National Institutes of Health, Bethesda) [2, 25]. One layer of voxels at the junction of the implant and bone were excluded to measure BICs. In addition, the other 2D μCT slices were chosen at interval of 45°, 90° and 135° with the representative histological section by the Dataview Software, and processed in the same procedures as above to calculate BIC (Figs. 1 and 2). Furthermore, the average of the BIC measured at four sites of the implant was calculated in the group which has strong correlation between 2D μCT and histology.

### Histomorphometry analysis

The specimens were fixed in the 10% formalin for 5 days and dehydrated in 100% alcohol. The specimens then were infiltrated in the chemical curing resin (methacrylate-based) and embedded with benzoyl peroxide. The blocks were cut along the longitudinal axis of the implant using EXAKT BS-3000 N (Norderstedt, Germany) and sectioned at 4 μm thickness along the sagittal plane through the center of the implant. The sections have performed the Hematoxylin and Eosin staining (H&E). The bone formation around the implant threads was observed with light microscopy (Olympus, Japan), and the four grooves identical to the 2D μCT slices were selected to calculate BIC using the Image J Software (Fig. 2) [25, 26].

### Statistical analysis

Statistical analysis was performed by SPSS (Version 23, IBM, USA). A paired t-test was used to compare the two methods of calculating BIC [6, 18]. The Pearson’s correlation coefficients (γ) was used to evaluate the agreement between the 2D μCT slices and histological sections [6, 18]. *P*-values < 0.05 were considered to be statistically significant.

## Results

A total of 18 implants of the three groups (6 implants per group) were collected to measure the BIC for assessing osseointegration. The BIC obtained by the representative histological sections was named BIC-His. The 2D μCT slice matching with the representative histological section was named 0°-μCT. The 2D μCT slices at a 45°, 90°, and 135° interval to the histological section were named 45°-μCT, 90°-μCT, and 135°-μCT, respectively.

**Fig. 3:**
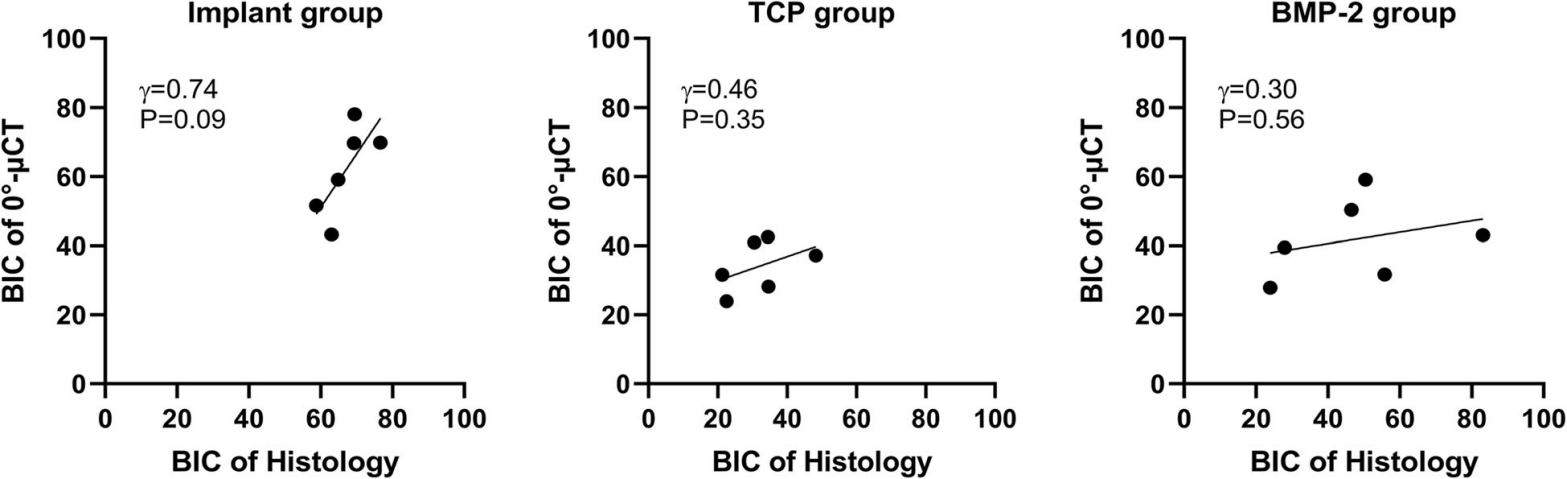
Correlation of BICs between the histological section and 0°-μCT. A strong correlation was presented in the implant group, a moderate correlation was shown in the TCP group, and a weak correlation was exhibited in the BMP-2 group. BIC: Bone-to-Implant Contact ratio; 0°-μCT: corresponding with the histological section; γ: Pearson’s correlation coefficient; P: *P*-value

There was no significant difference between the BICs obtained by histology and 2D μCT in each group (*P* > 0.05) (Table 1). The BICs of histological sections and BICs of 0°-μCT slices presented a strong correlation in the implant group (γ = 0.74, *P* = 0.09), a moderate correlation in the TCP group (γ = 0.46, *P* = 0.35), a weak correlation in the BMP-2 group (γ = 0.30, *P* = 0.56) (Table 1, Fig. 3).

**Table 1:** Comparison of BICs between histological sections and the corresponding micro-CT slices (0°-μCT) in each group

Group	N	BIC-Histology	BIC-0°-μCT	Mean difference	Pearson’s correlation (***P***-value)	***P***-value (Paired ***t***-test)
**Implant group**	6	67.02 ± 6.21	61.95 ± 13.05	5.07 ± 9.44	0.74 (0.09)	0.25
**TCP group**	6	31.94 ± 9.84*	34.08 ± 7.37*	−2.14 ± 9.17	0.46 (0.36)	0.59
**BMP-2 group**	6	47.97 ± 21.36	41.94 ± 11.65*	6.03 ± 21.01	0.30 (0.56)	0.51

Values are presented as mean ± standard deviation

*BIC* Bone-to-Implant Contact ratio, *μCT* 2D μCT slice, *N* number of specimens

*: *P*-value < 0.05, the significant difference compared with Implant group

Furthermore, the diverse correlations were shown between BICs-μCT at different sites (at 0°, 45°, 90°, and 135°) and BICs-His in the implant group. There were strong positive correlations of BICs between 2D μCT slices (at 0°, 45°, and 90°) and histological sections, and the moderate correlation was shown between 135°-μCT slices and histological sections (γ = 0.74, 0.81, 0.71, and 0.41; *P* = 0.09, 0.05, 0.11, and 0.42, respectively) (Table 2).

**Table 2:** Correlations of BICs between micro-CT slices at different sites and histological sections in the implant group

Comparison	N	BIC-μCT	BIC-Histology	Pearson’s correlation (γ)	***P***-value
**0°-μCT vs Histology**	6	61.95 ± 13.05	67.02 ± 6.21	0.74	0.09
**45°-μCT vs Histology**	6	60.24 ± 11.89		0.81	0.05
**90°-μCT vs Histology**	6	58.64 ± 11.27		0.71	0.11
**135°-μCT vs Histology**	6	58.33 ± 11.05		0.41	0.42

Values are presented as mean ± standard deviation

*BIC* Bone-to-Implant Contact ratio, *μCT* 2D μCT slice, *N* number of specimens

*P*-value < 0.05: statistically significance

**Fig. 4:**
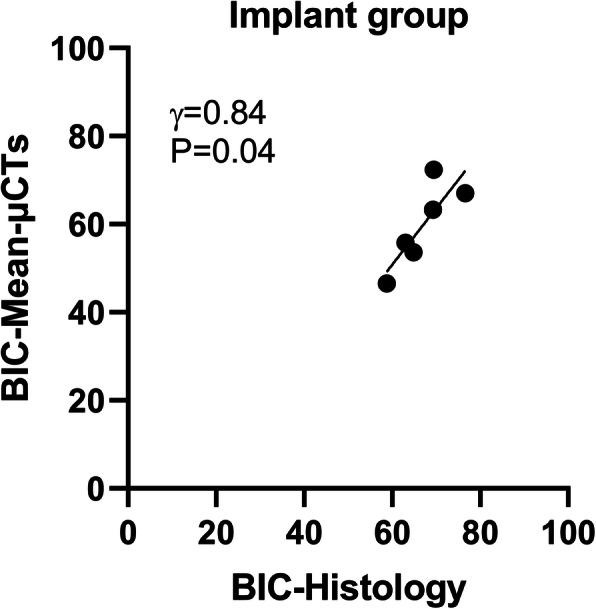
Significantly linear correlation between BIC-histology and BIC-μCT-mean in the implant group. BIC: Bone-to-Implant Contact ratio; BIC-μCT-mean: mean of BICs on the four μCT slices; γ: Pearson’s correlation coefficient; P: *P*-value

In addition, the mean of BICs at four different sites in each implant was calculate to assess the osseointegration of the overall implant, named BIC-Mean-μCTs. In the implant group, the relationship between BIC-Mean-μCTs and BICs-His has presented a significant linear correlation (γ = 0.84, *P* = 0.04, Fig. 4).

## Discussion

In the bone regeneration research, the specimen for histomorphometry must be obtained by sacrificing the experimental animals and could only offer the bone information of the limited locations. On the contrary, for the small animals (mouse and rat), micro-CT data could be achieved under anesthesia and provide a 2D μCT slice of any site. If the implant osseointegration could be speculated by the micro-CT slices, we could observe the osteogenesis at different time points through one animal and reduce the death of experimental animals. In addition, we consider that the BIC calculated by integrating bone information of several 2D μCT slices of different locations is more comprehensive to assess the osseointegration of the implant than BIC obtained by histomorphometry in part location.

In this study, we used an aluminum filter in scanning and adjust the correction functions of the analysis software to reduce the artifacts in the micro-CT reconstruction. The critical procedure in the present study is to select the threshold of newly formed bone in micro-CT analysis. The bone tissue is isolated from air, soft tissue (bone marrow), and metallic implant by the different specific threshold values. The ISO 50% method based on the grayscale histogram is commonly used to determine the bone threshold [18, 27]. However, the low-mineralized bone has a similar threshold value with bone marrow or soft tissue, and their threshold ranges unavoidably overlap with each other. Hence, the threshold of bone is chosen by the ISO 50% method has a deviation [27, 28]. Due to the high resolution, the low-mineralized bone can also be clearly distinguished from other tissues in the undecalcified histological slide. In order to improve the accuracy of the threshold, the histological sections were served as a reference to define the optimal threshold (min 60, max 90) for new bone in micro-CT analysis. Whereas, these operations cannot completely eliminate the interference of artifacts. Consequently, we excluded one layer of voxels at the junction between the bone and the thread to reduce the influence of artifacts in measuring BIC on the 2D μCT slice [11, 12].

In each group, BICs at the same sites measured by histomorphometry and corresponding 2D μCT presented the same trend and shown no significant difference between the two methods (Table 1). Moreover, a strong correlation (γ = 0.74) was found between BIC of 0°-μCT and BIC of histology in the implant group (Table 1, Fig. 3). Because of micro-CT scanned an object at a certain interval, it was difficult to find the 2D μCT slice that was exactly matching with the histological slice. Therefore, the correlation coefficient between the BICs measured by the two modalities was slightly reduced. In the TCP group and the BMP-2 group, the moderate and weak correlations were detected between BIC of 2D μCT and BIC of histology at the identical site (Fig. 3). We considered that the residual β-TCP granules affect the correlation coefficient in the TCP group and the BMP-2 group. The β-TCP granules in the hydrogel were not completely degraded within 4 weeks and could be distinguished from the bony tissue in the undecalcified histological section. Therefore, β-TCP was excluded in the BIC measurement for histomorphometry. On contrary, the β-TCP could not be distinguished from the bone in micro-CT and was identified as the new bone in the BIC measurement. Therefore, the BIC obtained by 2D μCT slice could be used to assess the implant osseointegration, when no bone graft-based substitutes and ceramic-based substitutes remain.

In the implant group, various correlation coefficients were shown between BICs of the 2D μCT at different sites and BICs of the representative histological sections (Table 2). It is demonstrated that the BIC acquired from histology of a specific location cannot illuminate the osseointegration of the whole implant or that at other sites. Therefore, we calculated the mean of BICs of the four μCT slices (at 0°, 45°, 90°, and 135°) of each implant to assess the osseointegration of the entire implant. A high correlation coefficient (γ = 0.84) was shown between BIC-Mean-μCTs and BIC-Histology (Fig. 4). However, the correlation will be altered when changing the site of histological section or increasing the number of μCT slices for calculating BIC-Mean-μCTs. And the more 2D μCT slices are used to calculate the mean of BICs, the closer the BIC-Mean-μCTs is to the osseointegration of the implant.

Some limitations exist in the present study. First, due to the small sample size, *P* values had biases in the present study. The second limitation is the small number of 2D μCT slices used for calculating the mean of BICs. Additionally, the method of minimizing artifacts still needs to be optimized. The BIC-Mean-μCTs supplies comprehensive information of the entire implant osseointegration, while the BIC-Histology offers an accurate assessment of the osseointegration at a specific site.

## Conclusion

The method that integrating bone information of several 2D μCT slices in different sites to measure BIC is feasible to assess the implant osseointegration, although the method needs further optimization.

## Data Availability

All data generated or analyzed in this study are included in this published article.
